# Principles of Optogenetic Methods and Their Application to Cardiac Experimental Systems

**DOI:** 10.3389/fphys.2019.01096

**Published:** 2019-09-11

**Authors:** Emily A. Ferenczi, Xiaoqiu Tan, Christopher L.-H. Huang

**Affiliations:** ^1^Department of Neurology, Massachusetts General Hospital and Brigham and Women’s Hospital, Harvard Medical School, Boston, MA, United States; ^2^Key Laboratory of Medical Electrophysiology, Ministry of Education and Medical Electrophysiological Key Laboratory of Sichuan Province, Institute of Cardiovascular Research, Southwest Medical University, Luzhou, China; ^3^Physiological Laboratory, University of Cambridge, Cambridge, United Kingdom; ^4^Department of Biochemistry, University of Cambridge, Cambridge, United Kingdom

**Keywords:** optogenetics, cardiac electrophysiology, opsin selection, light delivery, physiological readout, channelrhodopsin, halorhodopsin, archaerhodopsin

## Abstract

Optogenetic techniques permit studies of excitable tissue through genetically expressed light-gated microbial channels or pumps permitting transmembrane ion movement. Light activation of these proteins modulates cellular excitability with millisecond precision. This review summarizes optogenetic approaches, using examples from neurobiological applications, and then explores their application in cardiac electrophysiology. We review the *available opsins*, including depolarizing and hyperpolarizing variants, as well as modulators of G-protein coupled intracellular signaling. We discuss the biophysical properties that determine the ability of microbial opsins to evoke reliable, precise stimulation or silencing of electrophysiological activity. We also review *spectrally shifted* variants offering possibilities for enhanced depth of tissue penetration, combinatorial stimulation for targeting different cell subpopulations, or all-optical read-in and read-out studies. *Expression of the chosen optogenetic tool* in the cardiac cell of interest then requires, at the *single-cell level*, introduction of opsin-encoding genes by viral transduction, or coupling “spark cells” to primary cardiomyocytes or a stem-cell derived counterpart. At the *system-level*, this requires construction of transgenic mice expressing ChR2 in their cardiomyocytes, or *in vivo* injection (myocardial or systemic) of adenoviral expression systems. *Light delivery*, by laser or LED, with widespread or multipoint illumination, although relatively straightforward *in vitro* may be technically challenged by cardiac motion and light-scattering in biological tissue. *Physiological read outs* from cardiac optogenetic stimulation include single cell patch clamp recordings, multi-unit microarray recordings from cell monolayers or slices, and electrical recordings from isolated Langendorff perfused hearts. Optical readouts of specific cellular events, including ion transients, voltage changes or activity in biochemical signaling cascades, using small detecting molecules or genetically encoded sensors now offer powerful opportunities for all-optical control and monitoring of cellular activity. Use of optogenetics has expanded in cardiac physiology, mainly using optically controlled depolarizing ion channels to control heart rate and for optogenetic defibrillation. ChR2-expressing cardiomyocytes show normal baseline and active excitable membrane and Ca^2+^ signaling properties and are sensitive even to ~1 ms light pulses. They have been employed in studies of the intrinsic cardiac adrenergic system and of cardiac arrhythmic properties.

## Introduction

Optogenetics offers techniques to modulate the activity of excitable cells using light, in a genetically specified manner. The method harnesses microbial proteins, known as opsins, which are light-activated proteins (channels or pumps) that permit transmembrane movement of ions. There are two types of opsins: Type I (found in prokaryotes, algae, and fungi) and Type II (found in animals). The opsins used most commonly in optogenetics fall under the category of Type I opsins (although some Type II opsins are also used) and are referred to as rhodopsins, meaning that they contain both an opsin protein (with seven transmembrane domains) as well as a light-sensitive chromophore. When activated by light, opsins cause depolarization or hyperpolarization of the cell membrane, with resulting cellular excitation or silencing on a millisecond time scale. Opsins were first expressed in mammalian neurons in the mid-2000s; since then the optogenetic toolkit has vastly expanded to address a number of different experimental demands. Aided by the recent elucidation of the crystal structure of opsin proteins, opsin engineering has led to a wide array of available tools with different properties, including ion conductances, kinetics, light sensitivity, and activation spectra. Although initial applications of optogenetics were primarily within the field of neuroscience, the technique soon became relevant to other excitable tissues. In this review, we summarize existing optogenetic tools and approaches and discuss their suitability for cardiac experimental questions. We then review existing cardiac applications and discuss some recent examples in cardiac optogenetics that have yielded new insights into cardiac physiology and cardiac disease.

**Figure 1:**
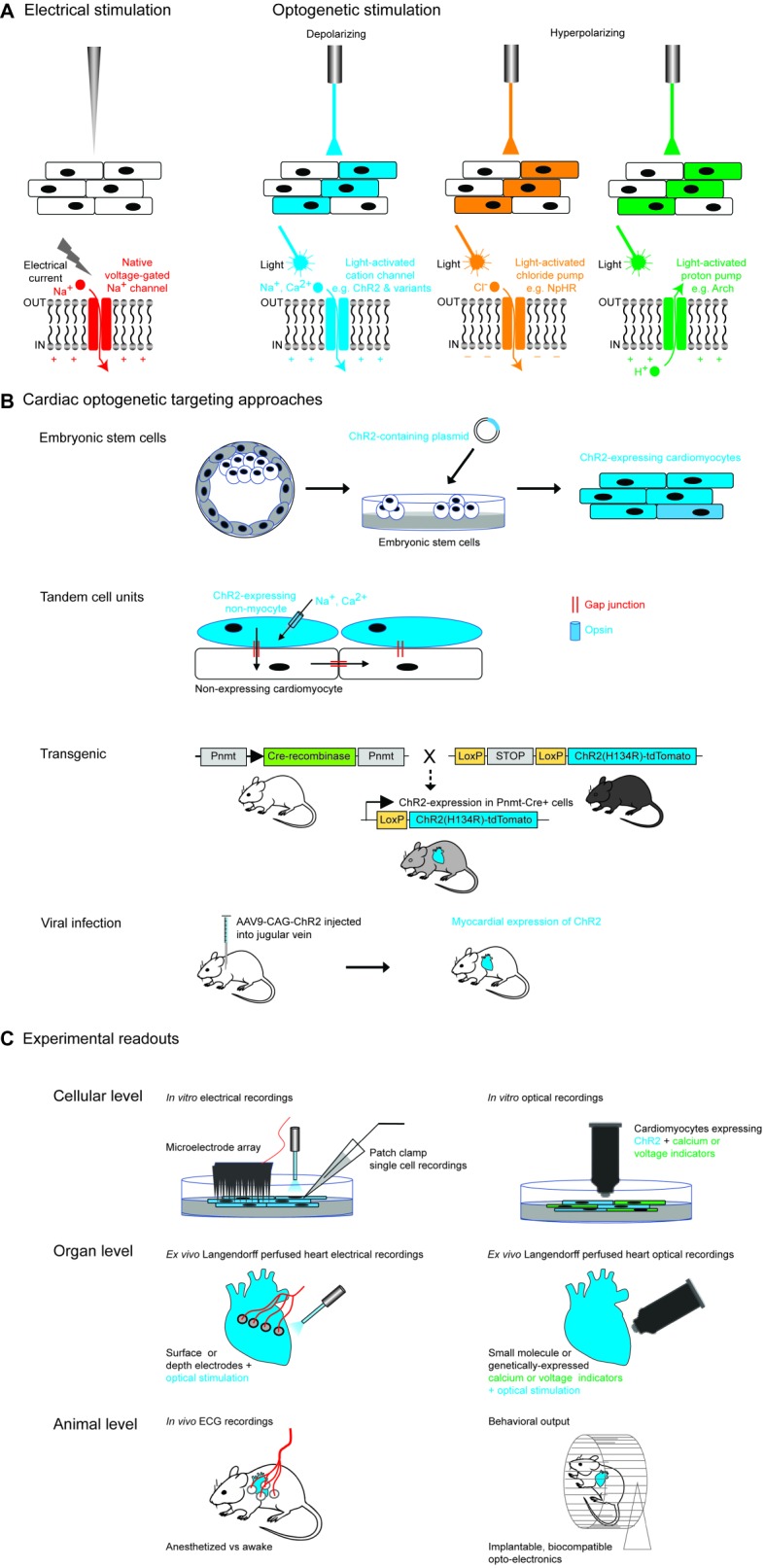
Optogenetic approaches in cardiac tissue. **(A)** Comparison of electrical and optogenetic stimulation paradigms, highlighting cellular specificity and bidirectionality of optogenetic techniques. **(B)** Examples of different methods of targeting opsins to cardiac tissue. Section on transgenic methods is adapted from Figure 1A from Wang et al. (2017). Section on viral infection describes the experimental approach used by Vogt et al. (2015). **(C)** Examples of different optogenetic read-outs for cardiac applications, ranging from *in vitro* to *in vivo* experimental set-ups.

Any optogenetic experiment involves several practical considerations: first, the properties of the chosen opsin tool and whether it is suited to the cellular physiology of interest and the experimental application. This includes the direction of the ionic current, as well as the nature of the transported ion(s), in addition to the kinetics and temporal precision of the tool, the amplitude of the evoked current (which in turn depends on a number of factors described below), and its spectral properties. The next consideration is the ability to specifically target (usually anatomically and/or genetically) the cell type of interest and deliver light into the experimental system of interest in a stable and spatially precise manner. Finally, the read-out of the system will depend on the experimental question being addressed; for example, this might be an electrophysiological recording, or a physiological imaging technique or an organ-level or behavioral output (Figure 1).

Studies of cardiac arrhythmias have traditionally depended upon electrical stimulation, which has the advantage of being temporally precise, but also carries several disadvantages including toxicity to the tissue, lack of cellular or anatomic specificity of the stimulation due to the rapid spread of current from the electrode tip, and lack of flexibility in terms of duration of stimulation (Bruegmann et al., 2010). The advent of optogenetics now allows temporally precise bidirectional modulation of cellular activity (using depolarizing and hyperpolarizing tools) (Figure 1A) and improved cellular specificity *via* genetic or developmental targeting methods and spatial specificity through the use of patterned illumination. The combination of optogenetic tools with optical activity sensors (e.g., calcium or voltage sensors) has also permitted realization of the highly desired “all-optical read-in and read-out” approach (Figure 1B).

The first cardiac optogenetic experiments were published in 2010, when two groups demonstrated light-induced modulation of function in mouse (Bruegmann et al., 2010) and zebrafish cardiomyocytes (Arrenberg et al., 2010). Since then, this approach has been applied to a wide range of cardiac experimental and translational questions including mechanisms of arrhythmia generation to optical defibrillation, arrhythmia termination, and pacing (Figure 1C).

## Selecting an Opsin for Cardiac Optogenetics: Directionality, Light Sensitivity, Kinetics, and Spectral Properties

### Depolarizing Tools

The first depolarizing rhodopsin-based tool to be expressed in mammalian neurons utilized a multicomponent system, in which *Drosophila* photoreceptor genes (rhodopsin + arrestin + G_q_α, a combination known as “chARGe”) were co-expressed in mammalian cultured hippocampal neurons (Zemelman et al., 2002). This system successfully drove light-activated neural firing and was the first proof-of-principle that rhodopsins could be used to modulate mammalian neural activity. One disadvantage of this multi-component system was its reliance on the exogenous delivery of retinal for rhodopsin photocycle function. In 2005, the first single-component microbial opsin, channelrhodopsin (ChR2), found in the algae *Chlamydomonas reinhardtii*, was expressed in mammalian neurons and elicited precise neural spiking in response to pulses of blue light (Boyden et al., 2005). In these early experiments, it was determined that sufficient retinal is present in vertebrate tissue for the ChR2 photocycle to function without the need for additional components. ChR2 is a non-selective cation channel, permeable to protons ≫ sodium ions > potassium ions ≫ calcium ions under physiological conditions (Deisseroth and Hegemann, 2017). When the channel pore is opened through a sequence of molecular changes that are initiated by photon absorption, the resulting net inward flow of positive ions results in a depolarizing photocurrent. This elicits an action potential when the excitable cell membrane reaches its firing threshold. Over the last 10 years, many naturally occurring depolarizing microbial rhodopsins have been found and genetically engineered mutant and chimeric rhodopsins have been developed, widely varying in terms of their channel kinetics, photocurrent magnitude, and spectral sensitivity (review: Deisseroth, 2015).

The ability of a depolarizing opsin to evoke precisely timed sequences of action potentials in response to pulses of light depends in part on the balance of two key properties – its light sensitivity and its channel kinetics, in combination with the physiological response of the cell to the photocurrent. The ability of a depolarizing rhodopsin to evoke precisely timed sequences of action potentials in response to pulses of light depends on the balance of several key properties. One is the photocurrent amplitude, which is in turn determined by light sensitivity (light intensity required to reach half maximal activation), the single-channel conductance, and the degree of channel expression in the cell membrane of interest. Other contributing factors include the channel kinetics as well as the cell’s physiological response to the photocurrent. Rhodopsins with higher light sensitivity require a lower light intensity to reach maximal photocurrent amplitude, and thus may drive depolarization more readily across a range of light intensities or across a larger volume of tissue (Mattis et al., 2011). However, in order to generate sustained action potential spiking in response to a long train of light pulses, channels should close quickly after the end of a light pulse to avoid sustained membrane depolarization and refractoriness; the kinetics of channel closure are described by the tau-off (*τ*_off_). In addition, the channels must have a low rate of desensitization and/or recover rapidly from the desensitized state. It has been demonstrated that there is a strong correlation between light sensitivity and *τ*_off_, suggesting that the light sensitivity of a membrane-bound rhodopsin population is mainly accounted for by channel off-kinetics, although differences in single channel intrinsic light sensitivity and conductance may explain deviations from the curve (e.g., in the case of the stable-step function opsins, see below). Others have noted that this suggests a theoretical trade-off between light sensitivity (volume of tissue activation) and channel kinetics (spiking precision) (Mattis et al., 2011). Fast channelrhodopsin mutants, such as the ChETA (ChR2/E123T) variants) have accelerated *τ*_off_ but lower light sensitivity; they are thus able to produce very precise trains of action potentials but require higher light intensities to do so (Gunaydin et al., 2010; Berndt et al., 2011). In contrast, mutant channelrhodopsins which are highly light sensitive (e.g., C1V1 variants, CatCh) are able to generate an initial action potential with high reliability but have slower *τ*_off_, higher plateau potentials, and thus a higher likelihood of spike failure over a train of light pulses. However, since light scatters within opaque tissue, opsins with higher light sensitivity may be more effective at generating spiking over a greater tissue volume (at increasing distance from the light source) (Broyles et al., 2018).

Newer highly light-sensitive bistable channelrhodopsin variants, known as “stable step function opsins” with *τ*_off_ of the order of ~30 min can provide stable subthreshold excitation over long time periods, even in the absence of ongoing light stimulation (Berndt et al., 2009; Yizhar et al., 2011b). Unlike the large photocurrents of fast opsins which drive precise action potentials, bistable variants instead produce small photocurrents that drive subthreshold depolarization, enhancing a cell’s excitability by increasing its sensitivity to physiologic excitatory inputs. These can be used for experimental paradigms that require persistent subthreshold excitation in the absence of ongoing light stimulation (Yizhar et al., 2011b; Ferenczi et al., 2016). The bistable step function opsins are activated by a brief pulse of blue light, but can also be switched off in a temporally precise manner by longer wavelengths of light.

The most frequently used opsin in cardiac tissue to date is ChR2_HR_ which is similar to the wild type ChR2 but has a point mutation that creates larger photocurrents at the expense of slightly slower *τ*_off_ (~20 ms). It is well expressed in cardiomyocytes and generates reliable action potentials in response to pulsed light (Bruegmann et al., 2010). However, when applying optogenetics to cardiac tissue, the specific properties of cardiomyocytes and non-myocytes should be considered. For example, for cardiomyocyte contraction, voltage-gated Ca^2+^ channels open secondary to an initial brief depolarizing input (e.g., from voltage-gated Na^+^ channel activation or an optogenetic depolarization), and the rise in intracellular Ca^2+^ through transmembrane influx and sarcoplasmic reticulum release generates the contractile force. A slow but sensitive opsin with large photocurrents and greater calcium ion permeability (such as CaTCh, Kleinlogel et al., 2011; Mattis et al., 2011; Bingen et al., 2014) may have the advantage of activating a larger volume of cardiac muscle, but risk leading to cellular calcium overload and could potentially be arrhythmogenic.

### Hyperpolarizing Tools

The first hyperpolarizing optogenetic tool was derived from the salt-deprived *Natromonas pharaonis* halorhodopsin (NpHR) (Han and Boyden, 2007; Zhang et al., 2007; Gradinaru et al., 2008). NpHR is a chloride pump activated by yellow light, which generates an inward chloride current at the resting somatic neuronal membrane potential. Proton pumps activated by green light, such as Arch/eArch (from *Halorubrum sodomense*), ArchT (from *Halorubrum* strain TP009), eBR (from *Halobacterium*), and Mac (from *Leptosphaeria maculans*) also generate hyperpolarizing currents from outwardly directed proton pumping (Chow et al., 2010). More recently, light-activated outwardly directed sodium pumps have also been shown to drive hyperpolarization and silence neural activity (Sudo et al., 2013; Tsunoda et al., 2017). Genetic modification of these hyperpolarizing tools through the addition of endoplasmic reticulum export motifs and membrane trafficking signals (Gradinaru et al., 2008; Mattis et al., 2011) has led to enhanced surface membrane expression and larger photocurrents, which has improved their efficacy in driving cell silencing. However, light-activated pumps have several disadvantages in electrically excitable tissue. Since they are not driven by naturally occurring electrochemical gradients, they are energetically inefficient compared to the light activated ion channels, with only one ion pumped per proton. Similarly, the pumps are not limited by physiological ionic gradients and may therefore be more likely to generate supra-physiological photocurrents that could be harmful to cell viability or produce paradoxical and unexpected cellular responses (Mattis et al., 2011; Ferenczi and Deisseroth, 2012). Therefore, over recent years, there has been a concerted effort to develop hyperpolarizing light activated ion channels through protein-structure guided channel engineering based on knowledge of the channelrhodopsin crystal structure (Kato et al., 2012; Wietek et al., 2014; Berndt and Deisseroth, 2015; Berndt et al., 2016). Through replacement of negatively charged amino acid residues within the channel pore of cation-selective channelrhodopsins, chloride-permeable variants with a negatively shifted reversal potential (~−60 mV in cultured mammalian neurons) can now be used for blue-light activated cellular silencing (iC1C2, iC++, ChloCs). Bistable highly light sensitive variants, analogous to the stable step function depolarizing opsins, SwiChR, SwiChR++, and SloChloC, have been engineered with off-kinetics of several seconds (in contrast to milliseconds for iC1C2), although the currents can be terminated rapidly by a pulse of red light when desired.

In 2015, naturally occurring anion-permeable light-activated ion channels (ACRs) were isolated from chlorophyte algae, *Guillardia theta* (GtACR1 and GtACR2) (Govorunova et al., 2015). Their crystal structures were also recently elucidated, opening many avenues for further understanding and modification of these channels (Kato et al., 2018; Kim et al., 2018). These chloride-permeable ion channels are characterized by their high intrinsic single-channel conductance, and as such have larger photocurrents and higher sensitivity than previously engineered variants and are able to efficiently silence neurons in culture. However, a major concern with both naturally occurring and engineered chloride channels is their tendency to induce antidromic spiking in axonal compartments, possibly as a result of differences in intracellular chloride concentration between the soma and the axon. Genetic modifications to improve membrane and somatic targeting have led to GtACR variants whose expression is restricted to the soma, allowing effective neuron silencing and decreased antidromic spiking in axons *in vivo* (Mahn et al., 2018). Despite these improvements, variability in intracellular chloride concentrations both within and between neurons remains a fundamental constraint of the chloride-permeable inhibitors. A more reliable and efficient form of inhibition would be through increased membrane permeability to potassium, and the search for a light-sensitive potassium channel still continues. Although no single-component potassium channel has yet been discovered or engineered, alternative strategies in which a plant photoreceptor LOV domain is fused to and controls a potassium channel (BLINK 1 and 2) has successfully been expressed in neurons to silence activity and modulate behavior in zebrafish and rodents (Alberio et al., 2018) and other two-component systems have been tested in cardiomyocytes (see below).

In cardiac optogenetics, Arrenberg et al. (2010) first used eNpHR in zebrafish hearts to drive myocardial cell hyperpolarization and cessation of myocardial contractions. Since then several groups have successfully suppressed cardiomyocyte activity using a variety of inhibitory optogenetic tools, including eNpHR (Abilez, 2012; Park et al., 2015b) and archaerhodopsin (Arch/ArchT) (Nussinovitch et al., 2014). Since then several groups have successfully suppressed cardiomyocyte activity using a variety of inhibitory optogenetic tools, including eNpHR (Abilez, 2012; Park et al., 2015b) and archaerhodopsin (Arch/ArchT) (Nussinovitch et al., 2014). ArchT-mediated cardiomyocyte hyperpolarization has also been used to terminate ventricular arrhythmias in the intact hearts of transgenic mice (Funken et al., 2019). As expected from the chloride reversal potential in cardiomyocytes, recent work (Kopton et al., 2017, 2018) has highlighted that cardiomyocytes in culture demonstrate excitation in response to light stimulation with both naturally occurring and engineered chloride channels. This can be explained by the fact that the reversal potential for chloride in the cardiomyocyte is more positive than the resting membrane potential, thus optically opening chloride channels tends to lead to membrane depolarization (Hiraoka, 1998). This property has been flexibly used in experimental preparations to allow either optical pacing of cardiac contractions in response to short pulses of light, or silencing of cardiomyocyte activity with prolonged activation through the induction of depolarization block (Kopton et al., 2018). Interestingly, other studies demonstrated an opposite effect on membrane potential, with ACRs eliciting large hyperpolarizing photocurrents with intracellular Cl^−^ concentrations adjusted by the patch pipette to 4 mM (Govorunova et al., 2016). The discrepancy between studies may be related to experimental preparations (e.g., intracellular chloride concentration) and developmental stage of the cardiomyocytes: the depolarizing effect was seen in cardiomyocytes isolated from rabbits age 9–10 weeks whereas hyperpolarization was seen in cultured neonatal rat cardiomyocytes. These findings also raise the importance of developing light-gated potassium channels, to more effectively stabilize the cardiac membrane potential at hyperpolarized resting potentials. Very recently, a two-component system consisting of a photo-activated adenylyl cyclase (PAC) fused to a small cyclic nucleotide-gated potassium channel (or split from each other by a self-cleaving 2A peptide), was expressed in cardiomyocytes and was found to reversibly silence cardiomyocyte activity *in vitro* and *in vivo* for tens of seconds (Bernal Sierra et al., 2018) (review: Entcheva, 2013).

### G-protein Modulators/Cell Signaling Modulators

Optogenetic tools are not only limited to ion channels, but also encompass a range of light-sensitive proteins that modulate intracellular signaling *via* G-protein coupled pathways. These can be naturally occurring or genetically engineered proteins. An example of a naturally occurring G-protein modulating opsin is melanopsin, which has been used in both neuroscience and cardiac research to generate sustained Gq activation (Koizumi et al., 2013; Beiert et al., 2014). In addition, the Opto-XRs are a family of engineered G-protein modulating opsins which have been designed to flexibly couple to different downstream signaling pathways (Airan et al., 2009). The first Opto-XRs were light-sensitive adrenergic receptors (ARs), known as Opto-α1AR and Opto-β2AR. These were shown to differentially modulate Gq and Gs signaling, respectively, with opposing effects on neural activity. A number of other chimeric tools have since been developed, including light-sensitive opioid receptors (OMOR) (rat rhodopsin coupled to the intracellular portion of the μ-opioid receptor) (Siuda et al., 2015), light-sensitive metabotropic glutamate receptors (melanopsin fused to intracellular components of mGluR6) (van Wyk et al., 2015), and light-sensitive serotonin receptors (melanopsin fused to intracellular loops of 5HT receptors) (McGregor et al., 2016). In cardiac physiology, a recent study has demonstrated the use of a jellyfish opsin (JellyOp) to activate Gs signaling in cardiomyocyte embryoid bodies and the intact heart (Makowka et al., 2019). An advantage of these tools is that for certain cell types (e.g., non-excitable cells such as glia) and synapses, they may provide a more physiological form of modulation, for example, mimicking natural neuromodulatory inputs. However, one disadvantage is that endogenous G-protein coupled pathways are dependent upon highly spatially localized machinery at the membrane for physiological propagation of the appropriate signal, which may not be in place with exogenous Opto-XR approaches. This has perhaps limited their widespread adoption over the last decade. Ongoing work is further refining and expanding this toolbox, which has promising translational applications, both for the understanding of intracellular signaling mechanisms as well as the development of pharmacologic therapeutic targets (Kato et al., 2012; Hegemann and Nagel, 2013; Deisseroth and Hegemann, 2017).

### Spectral Properties of Opsins

Alongside kinetics and photocurrent magnitude, much genetic engineering of opsins has focused on spectral tuning. The absorption spectrum of opsins is determined by the molecular and electrostatic environment of the chromophore within the protein, and knowledge of the crystal structure has provided key information to allow spectral tuning (by changing the energy of the ground state or excited state) through the modification of key residues of the rhodopsin molecules. This can be achieved by exchanging residues interacting with the retinal Schiff-base (e.g., counterion residues) or residues that influence the polarity along the retinal polyene chain (Yan et al., 1995; Kato et al., 2012; Hegemann and Nagel, 2013; Deisseroth and Hegemann, 2017). Wild-type channelrhodopsin has a peak activation spectrum of 470 nm (blue light activated), but naturally occurring and engineered variants have now been developed to cover a wide range of the visible spectrum from being even more blue-shifted (Govorunova et al., 2013) to red-shifted (Zhang et al., 2008; Yizhar et al., 2011b; Lin et al., 2013; Klapoetke et al., 2014). The first of these red-shifted variants to be discovered, VChR1 is maximally activated by green light (~530 nm), but retains strong photocurrents in amber light (590 nm), a wavelength that produced no measurable depolarization in ChR2-expressing cells. Further engineering of opsin chimeras and targeted mutant variants has led to an array of red-shifted opsins with large photocurrents and fast off-kinetics [C1V1 variants, red-activatable ChR (ReaChR), Chrimson] (Yizhar et al., 2011b; Lin et al., 2013; Klapoetke et al., 2014; Gupta et al., 2019). Several blue-shifted rhodopsin variants have also been identified. For example, one variant from a freshwater green alga [*Scherffelia dubia* channelrhodopsin (sdChR)] was genetically modified to have a spectral activation peak of 460 nm (10 nm blue shifted relative to ChR2) as well as faster kinetics. This variant, known as CheRiff, was shown to have 9-fold higher light sensitivity than ChR2_HR_ and had minimal optical cross-talk with red-shifted voltage indicators (Hochbaum et al., 2014). *Platymonas subcordiformis* ChR (PsChR) (Govorunova et al., 2013) and *Tetraselmis striata* ChR (TsChR) (Klapoetke et al., 2014) are the most blue-shifted variants identified to date, with a spectral activation peak of ~445 nm, but so far they have not been used widely in optogenetic applications. It is important to note that the newer red-shifted variants (e.g., Chrimson) have greater proton permeability than other ChR2 variants, and their use has been associated with pronounced intracellular acidification (Vierock et al., 2017).

A major advantage of red-shifted variants is the possibility of combinatorial stimulation experiments using both blue light and yellow or red light to activate different cell populations within the same anatomical area, or for all-optical read-in and read-out methods, in which stimulation is combined with imaging techniques. One limitation of the early red-shifted options was that despite their red-shifted shoulder many retained some activity within the blue light range (Venkatachalam and Cohen, 2014), making it challenging to spectrally segregate optogenetic stimulation at blue wavelengths. The most recent red-shifted mutant opsin, Chrimson-SA, which was spectrally tuned based on understanding of the crystal structure of Chrimson, addresses this issue, with maximal activation at 605 nm (20 nm further red-shifted than the original Chrimson) and much smaller photocurrents at the blue end of the spectrum (Oda et al., 2018). It may now be feasible to spectrally segregate optogenetic stimulation at blue and red wavelengths, although careful titration of expression levels and light intensities will be required. For imaging purposes (as will be discussed further in the “Optical read-out” section), it is possible to stimulate with ChR2 in the blue light range and image cellular activity with red-shifted calcium indicators [e.g., RCaMP, jRGECO1a; (Dana et al., 2016)]. Now with the most-red shifted Chrimson (Chrimson-SA), it may also be possible to stimulate at red wavelengths and image at blue wavelengths, although again, careful titration must be performed, and the appropriate controls included. Such spectral constraints also apply to the hyperpolarizing optogenetic tools (Wietek et al., 2017), which to date do not have such far red-shifted variants.

Another theoretical advantage of red-shifted spectra is volume and depth of tissue modulation. Since blue light is known to scatter more easily in opaque tissue such as the brain or cardiac tissue, red light has the potential to penetrate further with less scattering and thus may allow greater volume and depth of optogenetic modulation (Yizhar et al., 2011a,b), even through the intact skull (Lin et al., 2013).

## Targeting Opsins to Cardiac Tissue

Having chosen an optogenetic tool for a particular experiment, the next step is to express the tool in a cardiac cell of interest. There are a variety of methods to target specific cardiomyocyte or non-myocyte cells either *in vitro* or *in vivo* which we discuss below (Ambrosi et al., 2014) (Figure 1B).

### *In vitro* Targeting Methods

The first cardiac optogenetic study in mouse used developmental techniques with mouse embryonic stem cells (ESCs) to generate ChR2-expressing cardiomyocytes (Bruegmann et al., 2010). The ESCs were transfected with a plasmid encoding ChR2 using the β-actin promoter (CAG) to drive strong expression in cardiomyocytes. The ESC developed into embryoid bodies, within which cardiomyocytes were identified on the basis of their expression of the muscle specific protein α-actinin. Illumination of the embryoid bodies with pulsed blue light resulted in contraction of cells expressing ChR2. Bruegmann et al. (2010) also demonstrated that pulsed blue light could generate local electrical activity in a two-dimensional layer of purified ChR2-expressing ESC-derived cardiomyocytes, and that focal illumination at one site spread to other regions through the functional syncytium formed by the cell population. Prolonged illumination caused cessation of electrical activity, likely due to opsin-induced refractoriness of the illuminated area. Finally, in transgenic mice expressing ChR2-expressing ESCs, blue light illumination of the beating heart induced supraventricular or ventricular pacing depending on the site of illumination.

There have since been several additional reports of the use of embryonic stem cells to generate opsin-expressing cardiomyocytes. Beiert et al. (2014) transfected mouse embryonic stem cells with a plasmid containing a light-activated Gq-coupled receptor, melanopsin, to generate melanopsin-expressing cardiomyocytes in embryoid bodies (Beiert et al., 2014). Illumination with blue light resulted in faster beating of the embryoid bodies as well as irregular beating at high light intensities. Abilez et al. used lentiviral transfection of human-induced pluripotent stem cells (hiPSCs) to drive expression of both ChR2 as well as the first-generation inhibitory opsin, NpHR1.0 in hiPSC-derived cardiomyocytes to drive bidirectional control of cellular contractile activity (Abilez, 2012).

It is also possible to express optogenetic constructs in neonatal and adult primary ventricular cardiomyocytes using viral vector transduction (Ambrosi et al., 2015). Lentiviral vectors have been used to drive *in vitro* expression of ChR2 and NpHR in neonatal ventricular cardiomyocytes (Park et al., 2015b) and expression of channelrhodopsin variants such as CatCh in neonatal atrial cardiomyocytes (Bingen et al., 2014). Bernal Sierra et al. transduced cultured adult rabbit ventricular cardiomyocytes with an adenovirus vector containing a two-component cyclic nucleotide-activated potassium channel. These cells exhibited light-activated cardiomyocyte silencing for up to 5 days after isolation (Bernal Sierra et al., 2018).

At the single cell level, optical pacing has been accomplished in iPSc/ESC cardiomyocytes and both neonatal and adult cardiomyocytes. This is exemplified in tandem cell units (TCU) formed from non-excitable (e.g., HEK) cells (also known as “spark cells”) transfected to express a light-sensitive ion channel (ChR2) coupled through gap junctions to excitable cardiomyocytes (CM) to form an optically controllable functional TCU (Jia et al., 2011). Dual whole-cell patch clamp experiments demonstrated that TCUs formed from ChR2-expressing HEK cells successfully established coupling with neonatal rat or adult canine cardiomyocytes. Optical stimulation of ChR2-expressing HEK cells paced the flanking myocytes. The use of gap junction blockers demonstrated a requirement for functional gap junctions in such optical pacing. Using these functional TCUs, it was possible for the first time to combine optical excitation and optical imaging to record light-triggered muscle contractions and high-resolution propagation maps of light-triggered electrical waves. The latter were quantitatively indistinguishable from electrically triggered waves. Thus, illumination with 470 nm light activates ChR2, resulting in an inward current and cell depolarization in the illuminated non-excitable cell. Ionic exchange *via* gap junctions leads to cardiomyocyte excitation, and the resulting action potentials are then conducted throughout the functional syncytium of coupled cardiomyocytes.

Nussinovitch et al. demonstrated a similar TCU approach but this time using co-culture of ESC-derived cardiomyocytes with fibroblasts which were genetically engineered to simultaneously express both ChR2 and the red-shifted inhibitory opsin, ArchT. With this approach, they were able to generate bidirectional modulation, driving optogenetic pacing using blue light and optogenetic suppression of activity with red light (Nussinovitch et al., 2014). The advantage of this technique is that non-myocyte adult cardiac cells, such as fibroblasts, can survive *in vitro* for longer periods of time than primary cardiomyocytes.

The interactions between non-myocyte cardiac cells and cardiomyocytes have also been studied using this approach. For example, one study investigated the functional coupling between cardiac macrophages and cardiomyocytes in the AV node of mice, using a co-culture preparation. They demonstrated that the two cell types are electrically coupled *via* connexin 43 gap junctions in the AV node and exhibit synchronous membrane depolarization (Hulsmans et al., 2017). This approach then formed the basis for *in vivo* investigation of the functional role of this coupling on AV node conduction using optogenetic techniques (see below).

### *In vivo* and *ex vivo* Targeting Methods

An effective approach to generate opsin expression *in vivo* in a cell-type specific manner is through the generation of transgenic animals. This approach has been used to drive expression in both cardiomyocytes as well as cardiac non-myocytes. To date, optogenetic transgenic mice, zebrafish, and *Drosophila* have all been used in cardiac optogenetic experiments. One of the earliest cardiac optogenetic experiments was performed in transgenic zebrafish (Arrenberg et al., 2010). The Gal4/UAS genetic system was used to express the inhibitory NpHR or excitatory ChR2 in larval zebrafish cardiomyocytes. Illumination of the pacemaker region in the atrioventricular canal *in vivo* disrupted cardiac contractile activity and demonstrated that only a few cells (only three cells in some cases, 10–30 cells on average) were necessary for heart beat initiation.

In mice, several different cardiac transgenic models have been developed. One example is a transgenic mouse generated using the Cre-lox conditional genetic targeting system, in which mice expressing Cre-recombinase under the cardiac-specific α-myosin heavy chain (α-MyHC) promoter are crossed with mice which conditionally express ChR2_HR_ on exposure to Cre-recombinase (Ai27D mice). The resulting offspring expresses ChR2_HR_ specifically in cardiac tissues (Zaglia et al., 2015; Crocini et al., 2016; Scardigli et al., 2018). This approach has been used to investigate the propensity for cardiac tissue to generate focal ectopic electrical activity at different locations in the heart and under different physiological and pathological conditions, including cardiac ischaemia (Zaglia et al., 2015). It has also been used for temporally and spatially precise termination of ventricular arrhythmias under open loop and closed loop conditions (Crocini et al., 2016; Scardigli et al., 2018). Zaglia et al. (2015) also went one step further by driving ChR2 expression specifically in the Purkinje cell system, as opposed to expression throughout all cardiomyocytes. This was achieved by crossing connexin40-Cre mice with conditional ChR2-expressing mice, such that only the connexin-40 cells (present throughout the cardiac conducting system, including Purkinje cells and the atria) express ChR2. Using these two different genetic preparations, Zaglia et al. (2015) were able to compare the effects of optogenetic stimulation when applied to all cardiomyocytes versus when applied specifically to the conducting system. They found that the number of non-specifically targeted cardiomyocytes needed to initiate an ectopic focus was much greater (~1,300–1,800 cells) than the number of specifically targeted Purkinje fibers (~100 fibers).

Several studies have also reported on transgenic methods for optogenetic targeting of cardiac non-myocytes, such as sympathetic cardiac neurons, parasympathetic neurons, cardiac neuroendocrine cells, and cardiac macrophages. Cardiac sympathetic neurons were targeted by cross-breeding mice expressing Cre-recombinase under the catecholaminergic-specific promoter tyrosine hydroxylase (TH) with mice that express ChR2 in a Cre-dependent manner, resulting in mice that express ChR2 only in TH-expressing catecholaminergic cells. This transgenic model was used to study the specific role of cardiac sympathetic nerve activation on cardiac contraction and arrhythmia (Wengrowski et al., 2015; Prando et al., 2018). The role of parasympathetic input to the heart was studied in a similar manner, by crossing mice expressing Cre-recombinase under the choline acetyltransferase (ChAT) promoter (expressed by peripheral cholinergic parasympathetic neurons) with mice expressing Cre-dependent ChR2 (Moreno et al., 2019).

**Figure 2:**
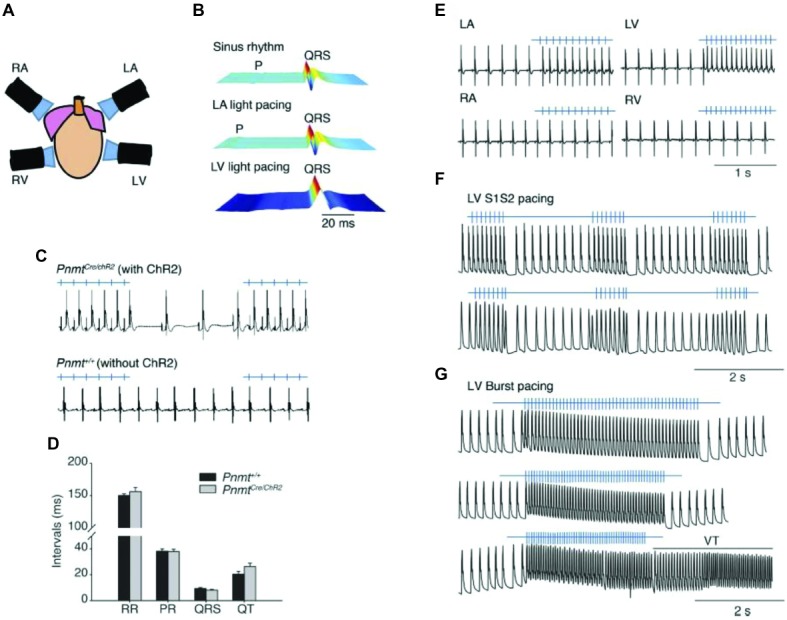
Control of heart rhythm and investigation of pro-arrhythmic markers in a *Pnmt-Cre/ChR2* mouse model expressing channelrhodopsin 2 (ChR2) permitting selective optogenetic stimulation of PdCM cells expressing phenylethanolamine n-methyltransferase (*Pnmt*). **(A)** Light pacing localized to 4 different regions of the heart. **(B)** ECG recording of a *Pnmt-Cre/ChR2* heart in intrinsic sinus rhythm (top) and following pacing applied to the left atrium (middle) and left ventricle (bottom panel). “P” denotes P wave in the ECG. **(C)** Light pulses delivered to the left atria of a *Pnmt-Cre/ChR2* but not a wild-type *Pnmt*+/+ heart evoking ECG spikes in a 1:1 manner. **(D)** Similar RR interval, PR interval, QRS interval and QT interval in Pnmt-Cre/ChR2 and Pnmt+/+ hearts. **(E)** Comparison of ECG entrainment with light stimulation applied to the left and right, atria and ventricles in a Pnmt-Cre/ChR2 heart. **(F,G)** Monophasic action potential recordings in the left ventricle of a Pnmt-Cre/ChR2 heart during programmed light stimulation delivered to the left ventricle in a S1S2 **(F)** or burst pacing protocol **(G)** [From Figure 3 of Wang et al. (2017)].

Another transgenic approach was used to drive ChR2 expression specifically in phenylethanolamine n-methyltransferase (*Pnmt*+) cells by crossing *Pnmt-Cre* mice with mice that express ChR2 in a Cre-dependent manner (Wang et al., 2017). The *Pnmt* gene encodes an enzyme converting noradrenaline to adrenaline, suggesting that early in development *Pnmt*+ cells may have neuroendocrine functions. In adult mice, these cells are predominantly located on the left side of the heart and have properties consistent with working cardiomyocytes. When illuminated with blue light, ChR2-expressing cardiomyocytes drove paced cardiac activity or generated arrhythmogenic activity, depending on the light-stimulation protocol, confirming that the activity of the subset of cardiomyocytes that have been *Pnmt*+ during development or underwent fusion with *Pnmt*+ cells was sufficient to control cardiac rhythm (Figure 2A). Light pulses delivered to the left atrium of a *PnmtCre/ChR2* (Figure 2B) but not wild-type *Pnmt*+/+ heart evoked ECG spikes (Figure 2C), with normal RR, PR, and QT intervals, and QRS duration (Figure 2D), a pacing effect not observed with pulsed right atrial or ventricular illumination (Figure 2E). Light pacing could be used to explore pro-arrhythmic properties under β-adrenergic stress using previously established stimulation protocols imposing extrasystolic stimuli progressively earlier after pacing trains of eight stimuli (S1) (Figure 2F), or burst pacing at variable (100–30 ms) or fixed (20 ms) cycle lengths (Figure 2G).

Similar approaches also been used to generate mice expressing hyperpolarizing optogenetic tools specifically in cardiomyocytes, by crossing *αMyHC-Cre* mice with mice that conditionally express ArchT on exposure to Cre-recombinase (Ai40D mice). This has been used to study hyperpolarization as a mechanism for ventricular arrhythmia termination (Funken et al., 2019).

Despite the flexibility of the Cre-LoxP systems, these approaches also have limitations, including leaky or incomplete Cre-recombination, which can result in off-target expression and compromise targeting specificity (Johnston et al., 2017). The non-inducible Cre-LoxP system also does not specify the cell type at the time of investigation, but rather highlights the developmental origin of Cre-positive cells or previous fusion events. To address some of these limitations, inducible Cre-LoxP expression systems have been developed to provide temporal control over the expression of Cre-recombinase and reduce leaky recombination events occurring early in development. This method was used to target cardiac macrophages (Hulsmans et al., 2017). In this study, mice expressing tamoxifen-inducible Cre driven by the *Cx3cr1* promoter (targeting cells of the mononuclear phagocytic lineage) were bred with mice expressing Cre-dependent ChR2, resulting in mice that express ChR2 specifically in AV node macrophages only after the administration of tamoxifen. This approach allowed the experimenters to causally assess the influence of AV node macrophage activation on atrial conduction *in vivo* and in a Langendorff-perfused heart preparation (Hulsmans et al., 2017). However, it is important to note the limitations of tamoxifen induction, which include variable induction strength across different tissues.

These studies represent the tip of the iceberg of genetically targeted cardiac cells. There are now a large number of cell specific promoters that may permit spatially constrained opsin expression in regions such as the sinus node, the atria, ventricles, conduction system, or progenitor cells. In addition, some genetic motifs may also permit subcellular targeting, for example, localizing the opsin to the plasma membrane, the sarcoplasmic reticulum or the mitochondria (Koopman et al., 2017). In parallel with the field of neuroscience, a number of groups have turned to viral vectors to drive opsin expression in cardiomyocytes and cardiac non-myocytes. Viral vector delivery using adeno-associated virus (AAV) is desirable for their safety and for potential application in humans, in contrast to transgenic or developmental methods. A disadvantage is that this approach will necessarily result in a smaller proportion of opsin-expressing cardiomyocytes, compared to other techniques. Vogt et al. (2015) demonstrated that opsin expression through intracardiac delivery of a viral vector can indeed be sufficient to modulate cardiac activity, and the expression has been shown to persist for over 12 months (Bruegmann et al., 2016). The AAV9 serotype has been shown to have good tropism for cardiomyocytes (Inagaki et al., 2006; Pacak et al., 2006) therefore was chosen for the optogenetic construct. The AAV9-CAG-ChR2_HR_-mCherry construct was injected systemically through the external jugular vein of wild-type mice. Expression of the fluorophore label, mCherry, was detectable after 4 weeks throughout the heart and evenly distributed across the ventricular wall. Dissociated ChR2-expressing cardiomyocytes showed robust inward photocurrents and action potential responses to short pulses of blue light. *In vivo*, blue light pulses could drive ventricular pacing (electrocardiographically recordable as broad QRS complexes without preceding P-waves) at 30–50 beats per minute above the resting heart rate in approximately three quarters of the tested hearts. Lack of *in vivo* optogenetic responses was likely the result of low construct expression, as demonstrated by lower mCherry fluorescent signal in the ventricles. The team calculated that the minimal percentage of expressing cells for optogenetic pacing was 30–40% and demonstrated as proof of principle that this percentage could be achieved through a systemic viral injection. One disadvantage of this method is off-target expression in other organs, for example, they also found mCherry-labeled muscle fibers in the diaphragm and a small number of hepatocytes. It may be possible to circumvent this with the use of cell-type specific promoters.

Viral vector delivery has also been used in other animal models. For example, one group used AAV2/9 to transfect cardiac sympathetic neurons in the left stellate ganglion of dogs with the hyperpolarizing tool ArchT (Yu et al., 2017). The authors demonstrated optogenetic suppression of myocardial-ischemia-induced ventricular arrhythmias in the dog heart, thus representing a first translational cardiac optogenetic application in a large, clinically relevant animal model.

## Light Delivery Systems

### Light Scattering in Cardiac Tissue

For *in vitro* cell culture or monolayer preparations, light delivery is relatively straight forward. For neurons, the light power density (number of incident photons per unit time per unit area multiplied by individual photon energy) required to elicit an action potential by wild type ChR2 has been estimated at ~1–5 mW/mm^2^ (Boyden et al., 2005), whereas in cultured myocytes, less than 1 mW/mm^2^ is required for optical pacing (Bruegmann et al., 2010). The reporting of light power density for optogenetic experiments has been discussed in detail elsewhere (Yizhar et al., 2011a). However, in optogenetic experiments in intact *in vivo* or *ex vivo* systems, light-scattering by biological tissue limits the even delivery of light through the tissue volume. In the brain, experiments using coherent light of different wavelengths through an optical fiber demonstrated that light in the brain scatters in a spherical distribution, rather than the conical distribution seen in saline, with longer wavelengths scattering less than shorter wavelengths (Yizhar et al., 2011a). In cardiac tissue, similarly careful measurements have been performed to determine the degree of attenuation of blue light across the myocardium (Zaglia et al., 2015). Analysis of the beam intensity profile over different depths of tissue from the optical fiber confirmed that over 90% of the total light intensity remained within a myocardial volume that was approximately cylindrical in shape. The depth and width of the cylinder could be modulated by the applied light intensity and the diameter of the optical fiber (Zaglia et al., 2015). These measurements were used to shape the volume of illuminated cardiac tissue in order to determine the minimal cardiac volume required to generate ectopic foci of activity.

### Mode of Light Delivery

Examples of light delivery systems can be found in published cardiac optogenetic experiments and recent reviews (Boyle et al., 2015, 2018a). Laser light is a commonly chosen light source for many applications, as the spectra can be closely matched to the activation peak of different opsins and the beams have low divergence and can be steered or patterned by mirrors or beam splitters on an optical table and coupled into optical fibers for high efficiency *in vivo* light delivery. Properties of lasers for optogenetics experiments are discussed in detail in earlier reviews (Yizhar et al., 2011a). However, LEDs have become increasingly attractive and lower cost light sources, with improvements in power output and spectral diversity over recent years. Disadvantages include their tendency to generate heat and the degree of divergence of LED beams, which lowers the coupling efficiency into optical fibers. Broadband light sources (e.g., arc lamp-based epifluorescence illuminators or light bulbs from video projectors (Arrenberg et al., 2010) can be coupled with shutters and spectral filters to generate spectrally flexible light sources. In *ex-vivo* preparations (e.g., Langendorff heart or *in situ* beating heart experiments), digital micromirror devices (DMDs) have also been used to generate user-defined light patterns to focus the light on different anatomical targets (Arrenberg et al., 2010; Scardigli et al., 2018).

For *in vivo* intracardiac light delivery, implantable microscopic inorganic LEDs (micro-ILEDs) either singly or as an array have been proposed. To overcome the issue of blue light scattering and increase the depth of light penetration, these micro-LEDs could be used in combination with red-light shifted opsins which tend to be less vulnerable to light scattering and achieve a greater penetration depth (such as C1V1 variants, ReaChR and Chrimson) (Yizhar et al., 2011b; Lin et al., 2013; Klapoetke et al., 2014; Boyle et al., 2015). The development of new biocompatible membranes has opened up the possibility for implantable, stretchable or injectable opto-electronic films or membranes (Kim et al., 2013; Liu et al., 2015) that could be applied to the epicardial or endocardial surface to provide widespread illumination across the myocardium or flexible multi-point illumination (Boyle et al., 2015). The development of new biocompatible membranes has opened up the possibility for implantable, stretchable or injectable opto-electronic films or membranes (Kim et al., 2013; Liu et al., 2015) that can be applied to the epicardial or endocardial surface to provide widespread illumination across the myocardium or flexible multi-point illumination (Boyle et al., 2015). Novel bio-optoelectronic devices have recently been developed to include soft biocompatible sensors and feedback systems for closed-loop control (Park et al., 2015a; Mickle et al., 2019). However, although these exciting advances have been demonstrated *in vivo* in the brain and other peripheral tissues (e.g., peripheral nerves, bladder), they have yet to be successfully applied to cardiac tissue for optical stimulation. Recently, a multi-LED probe was used for the first time to drive intramural optical stimulation of ChR2 in isolated murine hearts, demonstrating optical pacing as proof of principle (Ayub et al., 2018).

The use of two-photon illumination for optogenetic experiments in neurons is reviewed in detail elsewhere (Carrillo-Reid et al., 2017; Ronzitti et al., 2017; Alberio et al., 2018). Therefore here we will only briefly summarize its major advantages and challenges. Two-photon illumination allows for high spatial resolution stimulation in three dimensions, over a greater depth of penetration than traditional wide field one-photon illumination. In neuroscience, this has opened up new avenues for the investigation of the spatial complexity of neural activity and neural connectivity, for example allowing stimulation of multiple genetically defined individual neurons or their subcellular compartments such as axon terminals or dendrites (Packer et al., 2012). Several major technical challenges had to be overcome in order for two-photon optogenetics to be realized. These include the inherent low single channel conductivity and two-photon absorption properties of ChR2, such that with the small spot site of two-photon illumination, the degree of evoked membrane depolarization is insufficient to reach action potential threshold (Rickgauer and Tank, 2009). This has been addressed in several ways such as the use of chimeric opsins with enhanced two photon absorption (e.g., C1V1 variants) (Prakash et al., 2012) as well as adaptations of laser scanning techniques. By increasing the spot size (at the expense of axial resolution) in combination with laser scanning techniques to scan the two-photon spot rapidly over the surface of a single cell (~30 ms), it is possible to activate many rhodopsin molecules almost simultaneously and drive sufficient membrane depolarization to cause action potential initiation. An additional challenge is to scale this up in order to stimulate many multiple individual neurons simultaneously. One way to perform this is move the scanning beam quickly through space (raster scan), to sequentially target several different cells (e.g., achieved using scanning galvanometer mirrors, or acoustic optic deflectors). However, this is a relatively slow approach, especially if the goal is to target tens to hundreds of neurons simultaneously. An alternative approach is to generate patterned light illumination to direct light to multiple cells at once. This can be achieved with a digital mirror microarray device or more recently, digital holographic methods using a spatial light modulator (Ronzitti et al., 2017; Yang and Yuste, 2018).

Two-photon optogenetics also helps to circumvent challenges of spectral combinatorial approaches. For example, it permits the spatial segregation of optogenetic stimulation light from optical imaging light, such that one population of cells could be modulated optogenetically at the same time that the activity of a different but spatially intermingled population of cells is monitored with genetically encoded fluorescent activity sensors [e.g., GCaMP (Packer et al., 2015; Ronzitti et al., 2017)]. This is done most reliably with minimal cross-talk between the spectral channels when blue-shifted opsins are combined with red-shifted activity sensors (Forli et al., 2018) (see “Optical read out” section below). In additional, two-photon stimulation has the further advantage of shifting the excitation light to the near-infrared end of the spectrum, allowing deeper penetration into tissue. Successful two-photon optogenetic stimulation has now been demonstrated with a wide panel of engineered depolarizing, hyperpolarizing, and bistable variants, further increasing the range of experimental possibilities (Prakash et al., 2012).

In cardiac experiments, two-photon stimulation thus presents an even greater challenge. It is unlikely that the small stimulation volumes generated by two photon techniques will be sufficient to pace the well-coupled syncytium of cardiomyocytes with low input resistance (from Kir2.1 potassium currents). So far, two photon approaches for optogenetic stimulation have not been shown to work in the heart, although they may be used for high resolution imaging purposes.

## Cardiac Readouts

An essential component of any optogenetic experiment is to monitor the response of the experimental set-up to the applied optogenetic modulation, also known as a “read out.” This can occur at many levels of the biological hierarchy – from single cell or tissue, to whole organ or organism responses (Figure 1C).

### Electrophysiological Readouts

Electrical recordings represent a major type of readout of cellular and tissue response to stimulation. Depending on the experimental question, different readouts may focus on single cell activity using patch clamp electrophysiology (Bruegmann et al., 2010), multi-unit activity using micro-array recordings in cell monolayers or slices (Hofmann et al., 2010; Nussinovitch et al., 2014), whole organ activity in an *ex vivo* Langendorff preparation with depth or surface electrodes, or *in vivo* in an anesthetized or awake animal using ECG recordings (Bruegmann et al., 2010; Vogt et al., 2015; Wang et al., 2017). Electrical readouts have the advantage of high temporal resolution and for single cells provide a rich biophysical readout. However, for larger scale recordings in monolayers or whole organs, the spatial resolution and extent of electrical recordings are limited by the number and distribution of electrode contacts. Novel engineered electrodes and electrode arrays have attempted to overcome this limitation, such as grid electrodes and the new neuropixel probe which is of great interest to the neuroscience community (Steinmetz et al., 2018). However, this sampling limitation, along with the invasive nature of many types of electrical recordings, has also led to the development of alternative optical imaging approaches.

### Optical Readouts

A major goal in both neuroscience and cardiac research is to non-invasively read out cellular activity at single cell resolution over a wide area. To this end, much effort has focused on developing “all-optical approaches” for simultaneous optogenetic stimulation and recording of cellular activity. For a detailed description of the range of such experimental approaches, please see comprehensive review by O’Shea et al. (2019). Great strides have been made in this direction with the development of new genetically encoded optical sensors (Koopman et al., 2017). These sensors produce a fluorescent signal in response to specific cellular events, such as ion transients, voltage changes, or activation of biochemical signaling cascades. These have been reviewed in depth elsewhere (e.g., (Entcheva and Bub, 2016; Koopman et al., 2017; Deo and Lavis, 2018) and so here we will simply summarize the major categories of sensors that have been used or are of potential use in cardiac optogenetics.

The most widely used ion sensors are calcium sensors, although magnesium, chloride, and hydrogen ion sensors have also been described. Calcium sensors can be divided into two broad types – calcium-sensitive dyes (small molecule calcium chelators) and the more recently developed genetically encoded calcium sensors (GECIs). All GECIs contain a calcium-binding domain fused to either a single or two fluorescent proteins (FRET-based fluorescence). The single fluorescent protein sensors, GCaMPs, are now widely used in neuroscience due to their high signal to noise ratio and fast kinetics; however, unlike the FRET-based sensors (e.g., cameleon), they do not provide a ratiometric signal which can result in confounds during myocardial cell contraction. Despite this, GCaMPs have been used to record calcium transients in cardiomyocytes *in vivo* (Tallini et al., 2006), even at subcellular resolution [e.g., nanosparks (Shang et al., 2014)]. Initially, the GCaMP sensors had predominantly green emission spectra due to their GFP-based fluorescence, but newer versions with red-shifted emission spectra, including RCaMPs, R-GECO (Dana et al., 2016), and near-infrared-GECO (NIR-GECO) (Qian et al., 2019) have now been developed, which are important for combination with optogenetic tools. Despite extensive work on spectral tuning of different opsins, most optogenetic tools retain at least some activity in the blue end of the spectrum, making it difficult for them to be reliably combined with blue light-activated sensors. It is also important to note that there is potential cardiotoxicity from calcium indicators, for example, these have been shown to produce hypertrophy of the myocardium when used chronically (Yang et al., 2018) and various reports have suggested that GCaMPs may interfere with calcium channel dynamics, calcium buffering, and signaling (Steinmetz et al., 2017). To date, the field of cardiac optogenetics has largely opted to use the calcium-sensitive small molecule dyes. For example, Jia et al. (2011) first used the calcium dye Rhod-4 in a cardiac cell syncytium to image cardiomyocyte-mediated wave propagation triggered by optogenetic stimulation by ChR2. Recently, combinatorial viral constructs containing a blue light-activated opsin (in this case ChETA_TC_) and red-shifted genetically encoded calcium indicators (R-GECO) have been successfully used for optical stimulation and recording in primary ventricular cardiomyocytes (Chang et al., 2017).

Another desirable readout is optical measurement of membrane voltage, as opposed to a surrogate cellular response such as calcium transients. To date, the most frequently used optical voltage sensors in cardiac optogenetics are red-shifted voltage sensitive dyes. These have been used to map action potential propagation as well as emergent properties such as excitation waves in cardiac tissue with high temporal resolution (Burton et al., 2015; Entcheva and Bub, 2016; Feola et al., 2017). The red-shifted dyes have excitation wavelengths of >600 nm, ensuring minimal concomitant activation of channelrhodopsin or other blue-spectrum opsins. This all-optical approach was first performed by Park et al. (2015b) who demonstrated bidirectional voltage responses across a cardiomyocyte monolayer in response to stimulation by ChR2 or silencing by the inhibitory opsin eNpHR3.0. For this, they used Pittsburgh-I (PGH1) which is a voltage-sensitive dye with spectral properties highly suited for combination with blue-light activated optogenetic tools (excitation peak 608 nm, emission peak 880 nm) (Salama et al., 2005). Since then, multiple groups have employed voltage-sensitive dye imaging techniques in the Langendorff perfused heart (Zaglia et al., 2015; Crocini et al., 2016). Voltage sensitive dyes can also be used in combination with other small-molecule dyes – for example, Wang et al. (2017) used both calcium- (rhod-2) and voltage-sensitive (RH237) dyes to map calcium and voltage changes across atrial and ventricular tissue in a Langendorff heart preparation. They thus confirmed that the spread of excitation in response to optogenetic stimulation of adrenergic Pnmt-derived cardiomyocytes was comparable to that produced by conventional electrical stimulation (Wang et al., 2017). Closed loop systems are highly desirable for interventional applications. Scardigli et al. (2018) were the first to devise an all-optical closed-loop system, expressing ChR2 in transgenic mouse hearts under the cardiomyocyte-specific promoter alpha-MyHC and imaging action potential propagation in Langendorff-perfused hearts with the red-shifted voltage-sensitive dye (di-4-ANBDQPQ) (Scardigli et al., 2018). Using real-time image analysis, they used voltage responses in the ventricles or atria to trigger optogenetic stimulation to restore ventricular responses after AV block or interrupt re-entrant circuit arrhythmias. Finally, a key advantage of such all optical approaches is the capability of observing and manipulating emerging properties of excitable tissues, such as excitation waves (Burton et al., 2015; Entcheva and Bub, 2016; Feola et al., 2017).

Genetically encoded voltage indicators (GEVIs) have also seen great improvement over recent years. There are several different types of GEVI, including voltage-sensing fluorescent proteins (VSFP) (FRET-based or single fluorescent proteins) as well as opsin-based sensors. The proton-pumping microbial opsins such as archaerhodopsin (Arch), *L. maculans* (MAC), and subsequently engineered non-pumping mutants, have an endogenous fluorescence that is modulated by changes in local membrane voltage thus permitting voltage sensing with optical resolution of single action potentials (Kralj et al., 2012; Gong et al., 2013; Maclaurin et al., 2013; Yang and St-Pierre, 2016). Much work focuses on continuing to improve VSFP and rhodopsin-based GEVIs in terms of signal to noise ratio, temporal resolution and toxicity/off-target effects. Only very recently was a red-shifted GEVI developed with bright enough fluorescence to enable voltage monitoring in combination with optogenetic stimulation (Yi et al., 2018). In the heart, VSFPs have been used to monitor membrane voltage and action potential propagation in cardiac myocytes *in vitro*, *in vivo*, and ex vivo (Liao et al., 2015) as well as in cardiac non-myocytes (Quinn et al., 2016).

Finally, there are a number of optical sensors designed to detect changes in activity in biochemical transduction cascades, for example, calmodulin-CAMKII/calcineurin, cAMP/PKA, and cGMP/PKG activity. These biological processes and thus their sensors act over a slower time course than ion and voltage sensors. A number of FRET-based and genetically encoded sensors are available and have been used in combination with optogenetic stimulation in neurons [e.g., (Harada et al., 2017); reviewed in (Spangler and Bruchas, 2017)] but to date have not yet been combined with optogenetics in cardiac tissue. These sensors will be particularly useful for studying and testing longer-term modulatory effects of cellular and subcellular optogenetic activation in specific cardiac cell types (Koopman et al., 2017).

## Examples of Cardiac Applications of Optogenetic Techniques

Emerging optogenetic techniques provide unprecedented opportunities to study cardiac physiology particularly with the capacity to deliver optogenetic control with spatiotemporal resolution and cell type specific precision. Over the last decade, there has been an explosion in the use of optogenetics in cardiac physiology, with applications ranging from investigating action potential propagation across cardiomyocyte monolayers to arrhythmia initiation and termination *in vivo*. Future endeavors, many of which are already in progress, fall under a wide range of areas.

### *In vitro* Properties of ChR2-Expressing Cardiomyocytes

Single ChR2-expressing ventricular cardiomyocytes can be isolated from adult transgenic mice (generated from ChR2-expressing embryonic stem cells) (Bruegmann et al., 2010). These have been used to characterize the biophysical effects of ChR2 activation by application of blue light. The ChR2-EYFP fusion protein was localized to the ventricular cardiomyocyte cell membrane and t-tubular system, and ChR2-expressing cells showed normal resting membrane potentials, membrane resistances, and action potential durations relative to controls. In both ventricular and atrial cardiomyocytes, light application induced typical ChR2 currents measured by patch clamp with decay time constants similar to those reported elsewhere (Nagel et al., 2005) (~20 ms) and shorter than typical cardiomyocyte refractory periods. Action potentials could be elicited with even ~1 ms duration light pulses. Brief pulses of light reliably evoked action potentials. The action potentials resulting from brief light stimulation were accompanied by Ca^2+^ transients. Prolonged light stimulation induced action potentials followed by prolonged depolarizations. Such longer light stimulations resulted in prolonged elevations in intracellular levels of Ca^2+^ (Bruegmann et al., 2010).

ChR2 has also been used for precise local stimulation in two-dimensional *in vitro* cultures. The ChR2-expressing, ESC-derived cardiomyocytes were plated on multi-electrode arrays, where they formed two-dimensional functional syncytia of synchronously beating cells whose activity could be followed through local field potentials. Evoked electrical activity elicited by pulsed illumination spread to other regions and moving the illumination site correspondingly shifted the initial pacemaker site. Prolonged local illumination suppressed electrical activity in the illuminated area while sparing spontaneous activity in the non-illuminated areas consistent with this producing a sustained depolarization that would cause a local Na^+^ channel refractoriness.

### *In vivo* Electrophysiological Studies of ChR2-Murine Hearts

Illumination of ChR2 expressing cardiomyocytes permits introduction of novel stimulation patterns *in vitro* and *in vivo*, in investigations of physiological mechanisms involved in pacemaking or site-specific pro-arrhythmic effects of prolonged depolarizations and delayed afterdepolarizations. Thus, electrocardiographic studies of focal atrial ChR2 activation with pulsed blue illumination in anesthetized ChR2-EYFP expressing mice led to supraventricular pacing accompanied by prolonged (by ~50%) P-wave durations and PQ intervals (by ~20%) (Bruegmann et al., 2010). Ventricular illumination evoked ventricular extrasystolic beats with a delay of ~9 ms, prolonged QRS durations (by ~ 100%), and alterations in QRS waveforms that varied with the site of stimulation, reflecting action potential initiation at the level of the ventricular cardiomyocyte, rather than *via* the conducting pathways of the heart. An illumination area of 0.05 mm^2^ (with a blue light intensity of 7.2 mW mm^−2^), corresponding to ~50 epicardial myocytes in addition to cardiomyocytes in deeper layers, sufficed to induce pacing, but higher light intensities were required with smaller areas of stimulation. The latencies of electrocardiographic responses *in vivo* were similar to the observed delays from onset of illumination to action potential peak in cardiomyocytes *in vitro* (~10 ms). Finally, prolonged illumination likely leading to long-term ChR2 activation and sustained depolarizations in ventricular regions disturbed the regular pattern of sinus rhythm and generated spontaneous ventricular extra beats.

An alternative *in vivo* approach in genetically wild-type rats utilized adeno-associated virus (AAV) intramyocardial transgene delivery to express the *ChR2* transgene at one or more ventricular sites. Surface electrocardiographic measurements then revealed that focused blue light delivered *via* the optical fiber achieved efficient activation of the ventricle when applied at the apex (the site of ChR2-transgene delivery). It thus altered heart rates from a baseline sinus rhythm (~180 beats/min) to up to 300 beats/min during illumination. This did not occur when illumination was applied to remote myocardial areas in the same hearts or in animals injected with control AAV-CAG-GFP virus. Similar results were obtained in isolated perfused hearts. Other studies have demonstrated that ChR2 stimulation of cardiomyocytes is compatible with the use of long-wavelength voltage-sensitive dyes. These high resolution optical mapping studies confirmed that the ChR2-expressing cardiomyocytes were the source of pacemaker activity by demonstrating a shift in the site of the earliest ventricular activation – from the location of the conventional pacing electrode (at the side of the ventricles) to the site of ChR2-expression (at the apex) (Nussinovitch and Gepstein, 2015).

## Anti-Arrhythmic Interventions in ChR2-Murine Hearts

Applications of optogenetic techniques have been explored for their localizable, potentially translationally important, anti-arrhythmic effects (Bingen et al., 2014; Bruegmann et al., 2016, 2018; Crocini et al., 2016; Nyns et al., 2017; Funken et al., 2019). Classical electrical cardioversion including implantable cardioverter defibrillator (ICD) applies generalized electrical discharges to the whole heart without reference to particular re-entrant conduction pathways. Its resulting shocks cause pain, myocardium damage, and chest muscle contractures, with accompanying psychological discomfort. Optical defibrillation has the potential to develop and test bespoke effective therapeutic stimulation using much lower energy. This may need to address challenges such as potentially being less consistent than electrical defibrillation, due to variability in optogenetic expression whether between subjects and within a single heart, concerns about translatability of the required gene therapy to humans. There are also the technical issues relating to heating from and cumbersome nature of the light source and other hardware difficulties.

At the level of *in vitro* cellular preparations, anti-arrhythmic effects of light were explored in neonatal rat atrial cardiomyocyte monolayers transduced with lentiviral vectors encoding light-activated Ca^2+^-translocating channelrhodopsin (CatCh-eYFP) or with eYFP controls. Light pulses (duration 10 ms, wavelength 470 nm) then triggered action potentials only in the CatCh-expressing cultures. Burst pacing induced spiral waves rotating around functional cores, modeling atrial fibrillation. Optical and multi-electrode array (MEA) mapping assessed effects of prolonged (500 ms) light pulses activating CatCh. These terminated re-entry in all CatCh-expressing cultures but none of the controls. This took place through a uniform depolarization depressing overall excitability (Bingen et al., 2014).

In adult Wistar rats, systemically delivered cardiotropic adeno-associated virus vectors encoding the red-activatable channelrhodopsin (ReaChR) produced its global cardiac, cardiomyocyte-restricted, and transgene expression. This permitted ReaChR-mediated depolarization and pacing. Ninety-seven percent of ventricular monomorphic and 57% of polymorphic ventricular tachycardias (VT) induced by burst pacing could then be terminated by single 470-nm light pulses (1,000ms, 2.97mW/mm^2^) in Langendorff perfused hearts. This termination was preceded by prolongation of the action potential (by ~14 ms) and pharmacological interventions that shortened the action potential duration were observed to prevent arrhythmia termination (Nyns et al., 2017).

Epicardial optical stimulation through cardiac-expressed ChR2 also proved effective in terminating ventricular arrhythmias, whether in isolated perfused hearts from ChR2-transgenic or WT mice following adeno-associated virus-based ChR2 gene transfer. Illumination of the anteroseptal epicardium with blue light (470 nm) proved effective whether sustained ventricular arrhythmia was induced by electrical burst stimulation or S1-S2 protocols applied at the right ventricular base under conditions of reduced K^+^ (2 mM) and the presence of the K_ATP_ channel activator pinacidil (100 μM). Thus, uniform light-induced depolarization proved effective for cardiac defibrillation. Explorations of light duration, intensity, and size of illuminated area indicated a requirement for illumination lasting for at least the duration of an entire VT cycle (~80 ms), over an area > 15 mm^2^, and of intensity ~0.4–1 mW/mm^2^, suggesting a requirement for a depolarization of a critical portion of myocardium. Such findings extended to experimental infarct-related ST-elevation associated arrhythmia, following left anterior descending coronary artery ligation. Sustained ventricular arrhythmia following forced electrical pacing was terminated by a single light pulse. Concordant findings emerged from WT hearts after adeno-associated virus induced ChR2 gene transfer (Bruegmann et al., 2016).

Further optogenetic and imaging techniques simultaneously mapped and controlled electrical activity in intact ChR2-expressing transgenic mouse hearts made to show monomorphic VTs with spatially demonstrable slowed re-entrant conduction. Application of spatially defined stimulation patterns demonstrated that in contrast to a *single point* and *single line* stimulation, triple-line pattern optical stimulation at 10mW/mm^2^ successfully achieved cardioversion, doing so while confining the irradiated epicardium with a reduction of total irradiation energy (0.25 and 1 mJ, respectively) (Crocini et al., 2016).

Optogenetic techniques also proved experimentally effective in termination of atrial tachyarrhythmias in intact *ex* and *in vivo* hearts from transgenic and wild-type mice, respectively. Isolated ChR2 and pro-arrhythmic connexin 40, Cx-40-Ala96Ser expressing murine hearts perfused with low K^+^-Tyrode’s solution and an atrial K_ATP_-channel activator showed a pro-arrhythmic shortened atrial refractoriness and slowed atrial conduction. Atrial arrhythmia was terminated using epicardially focused blue light (470 nm, 0.4 mW/mm^2^) likely acting by light-induced block of electrical activity. This optical effect was reproduced in wild-type mice made to express ChR2 *via* adeno-associated virus (AAV) delivery (Bruegmann et al., 2018).

Finally, optogenetic methods permitted explorations of temporally and spatially controlled cell type selective induction of hyperpolarization as an approach to ventricular arrhythmia termination. These utilized mice with a cardiomyocyte-specific expression of the light-driven proton pump ArchT. The isolated cardiomyocytes showed light-induced outward currents causing membrane hyperpolarization. Isolated perfused hearts in which ventricular arrhythmias were induced by electrical stimulation under conditions of low [K^+^] and pinacidil showed a significant incidence of cardioversion upon illumination. *In situ* sharp electrode intracellular recordings showed an accompanying cardiomyocyte hyperpolarization. However, enhanced action potential upstroke rates upon illumination were accompanied by slowed conduction. Thus, the observed anti-arrhythmic effects might arise from accompanying increased electrical sink currents, suggestive of novel mechanisms by which the observed cardioversion took place. Such hypotheses might prompt further investigation and their possible application (Funken et al., 2019).

## Computational Analyses of Pro- and Anti-Arrhythmic Interventions Using ChR2-Murine Hearts

Computational studies have assessed the efficacy of optogenetic termination of both atrial tachycardia (AT) and VT through targeting specific cardiac regions if this was applied to human hearts. One study attempted to terminate re-entrant AT following rapid pacing by optogenetic stimulation of channelrhodopsin-2 (ChR2) expressed through gene delivery. This employed three patient-specific computational models reconstructed from late gadolinium-enhanced MRI scans (Boyle et al., 2018b). It compared efficacies of distributed endocardial illumination by multi-optrode grids and targeted illumination of the identified critical isthmus. The latter involved considerably smaller illuminated areas and input powers. AT termination rates for distributed illumination were low (<5% for short, 1/10 ms and ∼20% for longer, 100/1,000 ms pulses). Targeted illumination similarly achieved low outcomes for short (0% for 1/10 ms) pulses, but significantly better outcomes for longer stimuli (100 ms: 54% success; 1,000 ms: 90% success), exceeding the AT cycle length. Thus, targeted optogenetic stimulation based on analysis of AT morphology is a potential approach to atrial defibrillation. A computational exploration of optogenetic defibrillation for modeling infarct-related human VT suggested that red light illumination could terminate tachycardic episodes in diseased, ChR2-expressing hearts. This appeared to involve ChR2-mediated transmural depolarization of the myocardium inactivating voltage-dependent Na^+^ channels in cardiomyocytes through the ventricular myocardial wall interrupting wavefront propagation (Bruegmann et al., 2016).

## Translational Implications of Optical Pacing

Optogenetics offers potential alternatives to drug or electrotherapy for treating arrhythmia. Drug therapy for atrial fibrillation can have serious side effects even including proarrhythmic effects. Patients at risk from potentially fatal ventricular arrhythmias currently receive implantable defibrillators delivering electrical shocks terminating arrhythmias on demand. However, such electrical shock therapy is associated with discomfort and tissue damage.

Optical pacing is compatible with energy requirements that compare favorably with electric pacing and optical fibers may be more biocompatible than electrodes. Optical pacing could potentially simultaneously pace multiple sites with advantages for cardiac resynchronization therapy over biventricular electrical pacemakers (McAlister et al., 2007). Hypothetically, forced expression and subsequent activation of light-gated cation channels in cardiomyocytes might deliver a depolarizing force sufficient for atrial or ventricular defibrillation. There is also the possibility of utilizing optogenetic proteins generating hyperpolarizing current such as halorhodopsin or archaerhodopsin. Prolonged illumination producing sustained depolarization or hyperpolarization may successfully interrupt re-entrant electric activity thereby terminating arrhythmias. As indicated above, constant illumination of ChR2-expressing cardiomyocytes prolongs depolarization and refractoriness electrically silencing the illuminated areas (Bruegmann et al., 2010). However, such optogenetic approaches will require introduction of the ChR2 or other optogenetic probes either by a TCU approach (Jia et al., 2011) or through direct ChR2 cardiomyocyte expression (Bruegmann et al., 2010). They will involve establishing effective pacing strategies and stable pacemaker function without cell rejection or tumor formation. The clinical use of optical pacing may still be a distant prospect as it will need to establish superiority over existing implantable devices and surmounting technical challenges attached to using cardiac optogenetics *in vivo* (Sasse, 2011).

## Conclusions

In conclusion, the use of optogenetics in cardiology research has greatly accelerated over the last decade since the technique was first demonstrated as a proof of principle in zebrafish and mouse cardiomyocytes (Arrenberg et al., 2010; Bruegmann et al., 2010). The versatility of different optogenetic tools now allows extensive modulation of the morphology, direction, duration, and location of cellular membrane potential responses, and cardiac computational modeling will be important for developing and testing hypotheses regarding the impact of these different types of stimulation on the cardiac cycle (Boyle et al., 2013; Entcheva and Bub, 2016). Features of optogenetic tools, such as their versatility, bidirectionality, and high temporal, spatial and genetic precision confer a number of advantages over traditional electrical stimulation techniques. They also lend themselves to all-optical read-in/read-out experimental approaches, to allow modulation and monitoring of large numbers of individual cells in a minimally invasive manner. To date, these approaches have been applied to single myocardial cells and cell pairs, myocardial monolayers in culture, *ex vivo* Langendorff perfused hearts as well as *in vivo* hearts under anesthesia. Experiments have addressed fundamental questions about the physiology of the cardiac action potential and the spread of electrical activity across the heart as well as disease-focused questions such as mechanisms of arrhythmia initiation, propagation, and termination.

Ongoing work that will be shared with the neuroscience community will be the continued development and expansion of the optogenetic tool kit, and major goals include the development of red-shifted opsin that lacks a shoulder in the blue end of the spectrum and a single-component light activated potassium channel.

Future cardiac directions will need to focus on further optimization of optogenetic tools specifically for myocardial cells such as modification of kinetics of depolarization/hyperpolarization to better suit the cardiac action potential and modulation of specific ion permeability to minimize off-target effects of non-physiologic changes in intracellular ion concentration. Other work will need to focus on improving genetic methods to specifically target different myocardial cell types and improving engineering approaches to permit chronic *in vivo* light delivery with flexible illumination patterns and its combination with read out methods such as optical recordings for closed-loop stimulation paradigms. Improvements in optoelectronic device engineering with low power LED arrays for long-duration, multi-site stimulation, closed loop sensing and implantable or injectable flexible electronics that can mold to and adapt with the mobile myocardium will be necessary for cardiac optogenetics to become relevant for clinical applications. Finally, basic science and translational applications will continue to expand, for example, to include investigation of not only cellular but also subcellular mechanisms underlying myocardial excitability, contractility, rhythm generation and spread, in physiological as well as pathological states.

## Author Contributions

All three authors contributed to this review as a whole, with EF leading on the sections covering the principles and techniques bearing on optogenetic methods and XT and CH those aspects bearing on their cardiac application. Repeated revisions of the text were performed by EF and CH.

### Conflict of Interest Statement

The authors declare that the research was conducted in the absence of any commercial or financial relationships that could be construed as a potential conflict of interest.
